# Current recommendations for revascularization of non-infarct-related artery in patients presenting with ST-segment elevation myocardial infarction and multivessel disease

**DOI:** 10.3389/fcvm.2022.969060

**Published:** 2022-08-11

**Authors:** Korakoth Towashiraporn

**Affiliations:** Her Majesty Cardiac Center, Faculty of Medicine Siriraj Hospital, Mahidol University, Bangkok, Thailand

**Keywords:** ST-segment elevation myocardial infarction, multivessel disease, complete revascularization, percutaneous coronary intervention, infarct-related artery

## Abstract

ST-segment elevation myocardial infarction (STEMI) is a leading cause of morbidity and mortality worldwide. Immediate reperfusion therapy of the infarct-related artery (IRA) is the mainstay of treatment, either *via* primary percutaneous coronary intervention (PPCI) or thrombolytic therapy when PPCI is not feasible. Several studies have reported the incidence of multivessel disease (MVD) to be about 50% of total STEMI cases. This means that after successful PPCI of the IRA, residual lesion(s) of the non-IRA may persist. Unlike the atherosclerotic plaque of stable coronary artery disease, the residual obstructive lesion of the non-IRA contains a significantly higher prevalence of vulnerable plaques. Since these lesions are a strong predictor of acute coronary syndrome, if left untreated they are a possible cause of future adverse cardiovascular events. Percutaneous coronary intervention (PCI) of the obstructive lesion of the non-IRA to achieve complete revascularization (CR) is therefore preferable. Several major randomized controlled trials (RCTs) and meta-analyses demonstrated the clinical benefits of the CR strategy in the setting of STEMI with MVD, not only for enhancing survival but also for reducing unplanned revascularization. The CR strategy is now supported by recently published clinical practice guidelines. Nevertheless, the benefit of revascularization must be weighed against the risks from additional procedures.

## Introduction

Primary percutaneous coronary intervention of the IRA is the first-line reperfusion therapy for STEMI (1, 2). Approximately 50% of STEMI patients undergoing PPCI have concomitant MVD (3), which is strongly associated with future adverse clinical outcomes (4). MVD is defined as significant coronary artery stenosis of more than or equal to 70% of at least two major epicardial coronary arteries, or significant stenosis of one major epicardial vessel concurrent with at least 50% stenosis of the left main coronary artery (LMCA) (5, 6). An optical coherence tomography analysis reported a significantly higher prevalence of thin-cap fibroatheroma of obstructive non-IRA compared with non-obstructive lesions. The thin-cap fibroatheroma is considered a precursor for developing acute coronary syndrome (7). Therefore, “protective” PCI of the non-IRA aims that to achieve CR may be beneficial for preventing future adverse cardiovascular events. Nevertheless, the clinical benefits of immediate coronary artery revascularization should be weighed against the risks of longer procedure time, more contrast media, and more radiation from non-IRA PCI (8).

Moreover, PCI of the non-IRA lesion during acute events carry additional risks. During STEMI, circulating catecholamine levels may surge and cause significant coronary artery vasoconstriction. As a result, the non-IRA lesion may exhibit an exaggerated degree of coronary stenosis (9). This phenomenon may affect the physician's discretion regarding the revascularization strategy. In contrast, a substudy of the Reducing Micro Vascular Dysfunction in Revascularized STEMI Patients by Off-target Properties of Ticagrelor (**REDUCE-MVI**) trial reported a significantly higher fractional flow reserve (FFR) value of the non-IRA lesion measured immediately after PCI of the culprit-lesion compared to the one-month FFR (*P* = 0.001) (10). Consequently, the FFR value measured during acute events may be falsely negative.

This article reviews the current evidence and updated international recommendations of the revascularization strategy for STEMI with MVD patients who successfully undergo PPCI of the IRA.

## The major randomized controlled trials for complete revascularization in STEMI with MVD

There are several moderate-to-large scale RCTs and meta-analyses that demonstrate the clinical benefits of the CR strategy over IRA-only PCI.

### The RCTs of angiographic-guidance complete revascularization

The Preventive Angioplasty in Acute Myocardial Infarction (**PRAMI**) trial demonstrated the benefit of the preventive PCI strategy over IRA-only PCI (8). The investigators randomized 456 STEMI patients with MVD who successfully underwent PPCI into preventive PCI and the conservative treatment groups. The former group underwent angiographic guidance PCI immediately after successful PPCI of the IRA. Coronary stenosis of at least 50% was considered significant and preventive PCI was performed in these cases. Significant LMCA stenosis, chronic total occlusion (CTO), and cardiogenic shock (CS) were excluded from the study. After a median follow-up of 23 months, major adverse cardiac events (MACE) which consisted of cardiac death, non-fatal myocardial infarction (MI), and refractory angina were significantly lower in the preventive PCI group (hazard ratio [HR] 0.35; 95% confidence interval [CI] [0.21 to 0.58]; *P* < 0.001). The primary endpoint was mainly driven by the reduction in non-fatal MI (HR 0.32; 95% CI [0.13 to 0.75]; *P* = 0.009) and refractory angina (HR 0.35; 95% CI [0.18 to 0.69]; *P* = 0.002). The rate of cardiac death was similar between groups. It should be noted that the PRAMI trial was prematurely terminated because the interim analysis demonstrated a significant benefit from the preventive PCI strategy.

The Complete vs. Lesion-only Primary PCI (**CVLPRIT**) Trial reported that in-hospital CR was superior to the IRA-only PCI (11). After successful primary PCI, 296 patients were randomly assigned to the angiographic guidance CR or the culprit-lesion-only PCI group. The additional PCI could be immediately performed after culprit-lesion PCI or staged PCI during index hospitalization and before hospital discharge. The CVLPRIT trial also excluded CS patients, significant LMCA stenosis, and CTO. The CR strategy significantly reduced MACE (death, recurrent MI, heart failure, and repeat revascularization) at 12-months follow-up. Nevertheless, the individual endpoint was not statistically significant. The benefit of the CR strategy also extended to the longer follow-up period (mean of 5.6 years) which demonstrated a significant reduction of MACE (*P* = 0.0079) and the combined endpoints of death and MI (*P* = 0.0175) compared to the IRA-only PCI group (12).

### The RCTs of the fractional flow reserved-guidance complete revascularization

The Primary PCI in Patients With ST-elevation Myocardial Infarction and Multivessel Disease: Treatment of Culprit Lesion Only or Complete Revascularization (**DANAMI3-PRIMULTI**) Trial showed a significant benefit of FFR guidance CR over IRA-only PCI. This trial randomized 627 STEMI with MVD patients who successfully underwent IRA-PCI to further FFR-guided PCI of the non-IRA or to receive medication for the untreated lesions. Fractional flow reserve was performed within 2 days after primary PCI in all non-IRA lesions with more than 50% stenosis by visual estimation. An FFR value of ≤ 0.8 was considered to indicate the presence of significant ischemia. CS and STEMI caused by stent thrombosis were excluded. After a 12-month follow-up period, the FFR-guidance CR significantly reduced MACE (all-cause death, non-fatal MI, and ischemia-driven revascularization [IDN]) compared to the IRA-only PCI (HR 0.56; 95% CI [0.38–0.83]; *P* = 0.004). The primary outcome was strongly driven by the reduction of IDN (HR 0.31; 95% CI [0.18-0.53]; *P* < 0.0001) (13). However, in the cardiac magnetic resonance substudy of 280 patients, there were no significant differences in left ventricular (LV) ejection function (*P* = 0.39), LV infarct size (*P* = 0.62), or LV remodeling (*P* = 0.63) between the two groups (14).

The Comparison Between FFR Guided Revascularization vs. Conventional Strategy in Acute STEMI Patients With MVD (**COMPARE-ACUTE**) Trial compared the immediate FFR-guided CR vs. the IRA-only PCI. Eight hundred and eighty-five patients were randomized at a 1:2 ratio. In both groups, FFR was performed during the index procedure after successful primary PCI. An FFR value of ≤ 0.8 was considered physiologically significant. Staged PCI was conducted not more than 72 h after primary PCI. For the IRA-only group, the treating cardiologist reported the degree of coronary artery stenosis but was blinded to the FFR value. If the treating cardiologist decided to send the patient to undergo elective PCI within 45 days, this was not counted as an adverse event. Major adverse cardiac and cerebrovascular events (MACCE) were significantly lower in the acute FFR-guided CR group (HR 0.35; 95% CI [0.22–0.55]; *P* < 0.001). The result was driven by the reduction in repeat revascularization by PCI (HR 0.37; 95% CI [0.27–0.57]; *P* < 0.001). The rates of death from any cause and MI were similar (15).

### The RCTs of the combined angiographic or fractional flow reserved-guidance complete revascularization

The Complete vs. Culprit-Only Revascularization Strategies to Treat Multivessel Disease after Early PCI for STEMI (**COMPLETE**) Trial is the largest RCT to compare the CR strategy vs. IRA-only PCI. Four thousand and forty-one STEMI patients (3,738 underwent primary PCI and 303 were treated by pharmacoinvasive strategy) were randomized. Coronary artery stenosis of at least 70% was considered significant. For borderline lesions (50-69% stenosis), FFR was performed and a value <0.8 indicated significant ischemia. However, it should be noted that only 0.8% of the patients measured the FFR. The staged procedure was performed during the index admission or within 45 days after the index procedure. Among the enrolled population, approximately 0.2% had LM as the culprit lesion and 2% had CTO as the residual lesion. The primary dual antiplatelet therapy (DAPT) in the COMPLETE trial was aspirin and ticagrelor (or prasugrel) which reflect the current regimen of DAPT for STEMI patients underwent PPCI (16).

The COMPLETE trial had two co-primary endpoints. The first co-primary endpoint was combined CV death and MI, and the second co-primary endpoint was the combination of CV death, MI, and IDN. As compared to the IRA-only PCI, the CR strategy significant lowered the first (HR 0.74; 95% CI, 0.6–0.91; *P* = 0.004) and the secondary (HR 0.51; 95% CI, 0.43–0.61; *P* < 0.001) endpoints. For the individual endpoint, the CR strategy reduced the rate of MI, IDN, and unstable angina, while CV death and death from any cause were similar (17). In addition, the COMPLETE trial also demonstrated the benefit of CR across the reperfusion strategy, including primary PCI and pharmacoinvasive treatment (18). Because the majority of the patients in the COMPLETE trial (76–77%) had only one residual diseased vessel, the SYNTAX (Synergy between PCI with Taxus and Cardiac Surgery) score was relatively low compared to other studies (19). As a result, caution is needed when applying the results of the COMPLETE trial to daily practice.

### Meta-analyses of complete revascularization in STEMI with MVD

There are conflicting data derived from meta-analyses on the impact of the CR strategy on hard outcomes. Several meta-analyses have demonstrated a survival benefit of the CR approach for STEMI with MVD patients (20–23) and the reduction of MI (20, 22). Whereas, Osman et.al. did not report significant hard endpoints of the CR over culprit-only PCI including CV death (RR 0.78; 95% CI, 0.60–1.03; *P* = 0.08), MI (RR 0.73; 95% CI, 0.58–1.08; *P* = 0.08), and all-cause death (RR 0.90; 95% CI, 0.73–1.12; *P* = 0.36) (24). Nevertheless, all meta-analyses demonstrated a marked reduction of further unplanned revascularization in the CR strategy (20–24). Pavasini R et al. demonstrated that eight treatments are needed to prevent one unplanned revascularization (21). Regarding safety of the CR strategy, Ahmed et al. reported a non-significant difference in the rate of contrast-induced nephropathy (*P* = 0.152) and the risk of bleeding (*P* = 0.540) between CR and culprit-only PCI (20).

### The revascularization strategy for the STEMI patient with cardiogenic shock

STEMI patients with CS have a very high short-term morbidity and mortality rate (25, 26). Among CS complicating STEMI, approximately 80% had MVD (27). Immediate invasive strategies for reperfusion are the mainstay treatment among these patients (28). The Culprit Lesion Only PCI vs. Multivessel PCI in Cardiogenic Shock (**CULPRIT-SHOCK**) Trial compared IRA-only PCI vs. multivessel PCI in the setting of CS complicating acute myocardial infarction (AMI) patients. Seven hundred and six AMI with MVD patients were included in the study. About 60% of the population were diagnosed with STEMI. For the multivessel PCI group, the operator needed to PCI all major epicardial vessels of more than 2 mm. with at least 70% stenosis by visual estimation, including the revascularization of the CTO. The maximum contrast media allowed was 300 ml. For the IRA-only PCI, staged PCI could be performed if residual ischemia is persisted. The primary endpoint was the combination of all-cause death and severe renal failure requiring renal replacement therapy (RRT) at 30 days. Eighty-one percent of the immediate multivessel PCI group achieved CR after the index procedure and 17.4% of the IRA-only PCI group underwent staged revascularization. The incidence of MACE was significantly higher in the multivessel PCI group (55.4 vs. 45.9%; RR 0.83; 95% CI [0.71–0.96]; *P* = 0.01). The result was mainly driven by the rate of all-cause death in the multivessel PCI group (51.6 vs. 43.3%; RR 0.84; 95% [CI 0.72–0.98]; *P* = 0.03).

A meta-analysis that included 73,528 patients from 18 real-world observational studies demonstrated a lower rate of short-term renal failure (odds ratio [OR] 0.75; 95% CI [0.64–0.88]) and short-term stroke (OR 0.86; 95% CI [0.77–0.96]) among the culprit-lesion-only PCI group. Interestingly, the rate of long-term MI was lower in the multivessel PCI group (OR 1.12; 95% CI [1.03–1.22]). As a result, the authors suggested performing culprit-lesion-only PCI to avoid the short-term adverse events, followed by staged PCI for CR to reduce the risk of future MI (29).

### The revascularization for CTO as non-culprit lesion

The Evaluating Xience and Left Ventricular Function in Percutaneous Coronary Intervention on Occlusions After ST-Segment Elevation Myocardial Infarction (**EXPLORE**) Trial included 304 STEMI patients who successfully underwent PPCI of the infarct artery and had concomitant CTO lesion. This trial compared additional CTO revascularization within 1 week after PPCI (150 patients) to the conservative treatment (154 patients). The primary endpoints were the left ventricular ejection fraction (LVEF) and left ventricular end-diastolic volume (LVEDD) assessed by cardiac magnetic resonance imaging after 4 months. There were no statistically significant between the two study groups for both LVEF (*P* = 0.60) and LVEDD (*P* = 0.70). For the subgroup analysis, the CTO revascularization of the left anterior descending artery had significantly higher LVEF (47.2 ± 12.3% [CTO-PCI] versus 40.4 ± 11.9% [conservative group]; *P* = 0.02) (30).

The 2019 global expert consensus for CTO-PCI suggests confirming the presence of ischemia and viability in the myocardium before considering CTO-PCI (31). The 2021 American College of Cardiology (ACC)/ American Heart Association (AHA) Guideline for Coronary Artery Revascularization recommends the heart team approach before performing the CTO revascularization (32).

### Timing of staged revascularization for complete revascularization

In the subgroup analysis of the COMPLETE trial for the timing of the staged PCI during index hospitalization vs. after hospital discharge, the *P*-value for interaction was nonsignificant in both co-primary endpoints (*p for interaction* = 0.62 [the first co-primary endpoints] and 0.27 [the second co-primary endpoint]). This means that there was no significant difference regarding the timing of CR for the clinical outcomes. The staged procedure could either be conducted during the index hospitalization or selectively postponed after hospital discharge (17). Two meta-analyses also reported a non-significant impact of the timing of revascularization (immediate vs. staged) on cardiac death (20, 22) and the reduction of unplanned revascularization and MI (20).

However, a different meta-analysis reported a greater risk reduction for developing MACE with immediate non-IRA PCI over staged revascularization (relative risk reduction [RR] 0.40; 95% CI [0.32–0.5] and RR 0.69; 95% CI [0.54–0.89], respectively; *P for interaction* = 0.002) (24). Overall, the evidence suggests that the CR strategy should be done, whether immediately after primary PCI or as a staged procedure after hospital discharge. However, there are currently no large RCTs that address the optimal timing of the additional procedure.

The recently developed PRAISE (prediction of adverse events following an acute coronary syndrome) score which is based on machine learning demonstrates promising results for predicting the death, MI, and bleeding following an ACS (33). This means that this novel risk score should predict not only ischemic but also bleeding risks. As a result, the PRAISE score assists the physician to tailor the individualized antithrombotic regimen for ACS patients and potentially the appropriate timing for revascularization for high-risk patients.

### The guidance for revascularization

#### The intravascular imaging guidance for revascularization

Several clinical trials demonstrated the role of intravascular imaging in identifying high-risk coronary plaques that would gain clinical benefits from the preventive PCI strategy.

The Imaging Study in Patients With Unstable Atherosclerotic Lesions **(PROSPECT)** trial enrolled 697 ACS patients who underwent PCI and intravascular ultrasound (IVUS). The non-culprit lesions that contained the following features are associated with future adverse events: a minimal luminal area (MLA) of 4.0 mm^2^ or less (HR, 3.21; 95% CI [1.61–6.42]; *P* = 0.001) a plaque burden of at least 70% (HR, 5.03; 95% CI [2.51–10.11]; *P* < 0.001) or thin-cap fibroatheromas (HR, 3.35; 95% CI [1.77–6.36]; *P* < 0.001) (34).

Optical coherence tomography (OCT) is also useful to analyze vulnerable plaque. The prospective clinical trial conducted by Niccoli G et al. reported a worse prognosis for ACS patients with plaque rupture compared to those with intact fibrous caps (35). Montone RA et al. stated the presence of the macrophage infiltration assessed by OCT was associated with a higher incidence of MACE (36). Prati F et al. evaluates the untreated coronary artery using OCT and access the adverse clinical outcomes. The authors identify the high-risk lesions as followed: MLA <3.5 mm^2^ (HR 2.1; 95% CI [1.1–4.0]), lipid arc circumferential extension more than 180 degrees (HR 2.4; 95% CI [1.2–4.8]), fibrous cap thickness <75 μm (HR 4.7; 95% CI [2.4–9.0]), and OCT-defined macrophages (HR 2.7; 95% CI [1.2–6]) (37).

#### The physiological guidance of revascularization

The evidence regarding the utilization of physiologic studies (FFR or instantaneous wave-free ratio [iFR]) is mostly derived from stable coronary artery (CAD) patients. As a result, the data to support the role of FFR/iFR in the setting of ACS is still lacking (38).

The pool-analysis of the Instantaneous Wave-Free Ratio vs. Fractional Flow Reserve in Patients With Stable Angina Pectoris or Acute Coronary Syndrome **(iFR-SWEDEHEART)** and Functional Lesion Assessment of Intermediate Stenosis to Guide Revascularisation **(DEFINE-FLAIR)** reported a higher MACE rate of the deferral strategy in ACS patients compared to stable CAD (5.91% [ACS] vs. 3.64% [Stable CAD]); fully adjusted (HR: 0.61; 95% CI [0.38 to 0.99]; *P* = 0.04) (39).

A meta-analysis published in 2018 by Liou KP et al. that included 5,457 patients from 9 studies also reported a higher rate of MACE for deferral strategy using FFR in the ACS setting compared to stable CAD (17.6 vs 7.3 %; *p* = 0.004) (40). Therefore, in the setting of ACS, functional ischemic studies alone seem not to be appropriate for identifying which non-culprit lesions are safe to defer (38).

#### Angiographic-guided vs. FFR-guided revascularization

The current evidence suggests that angiographic-guided and FFR-guided revascularization provide similar clinical outcomes.

The FLOW Evaluation to Guide Revascularization in Multi-vessel ST-elevation Myocardial Infarction (**FLOWER-MI**) Trial did not show a benefit of angiographic-guided CR over the FFR-guided CR in STEMI with MVD patients at 12-month follow-up (HR 1.32, 95% CI [0.78-2.23]; *P* = 0.31) (41). The staged procedure (including angiographic-guided PCI and FR-guided PCI) could be performed during the index procedure or within 5 days and before hospital discharge. Coronary artery stenosis of at least 50% was considered significant. For the FFR group, FFR was measured in all lesions that had a degree of stenosis of at least 50%, and an FFR value of <0.8 was considered positive for ischemia.

A 2020 meta-analysis by Bainey KR et al. reported a non-significant interaction between angiographic-guided vs. the FFR-guided CR on hard endpoints such as all-cause mortality (*p for interaction* = 0.54), CV death (*p for interaction* = 0.73), MI (*p for interaction* = 0.68) (20) and combined CV death or MI (P for interaction = 0.52) (23).

**Figure 1:**
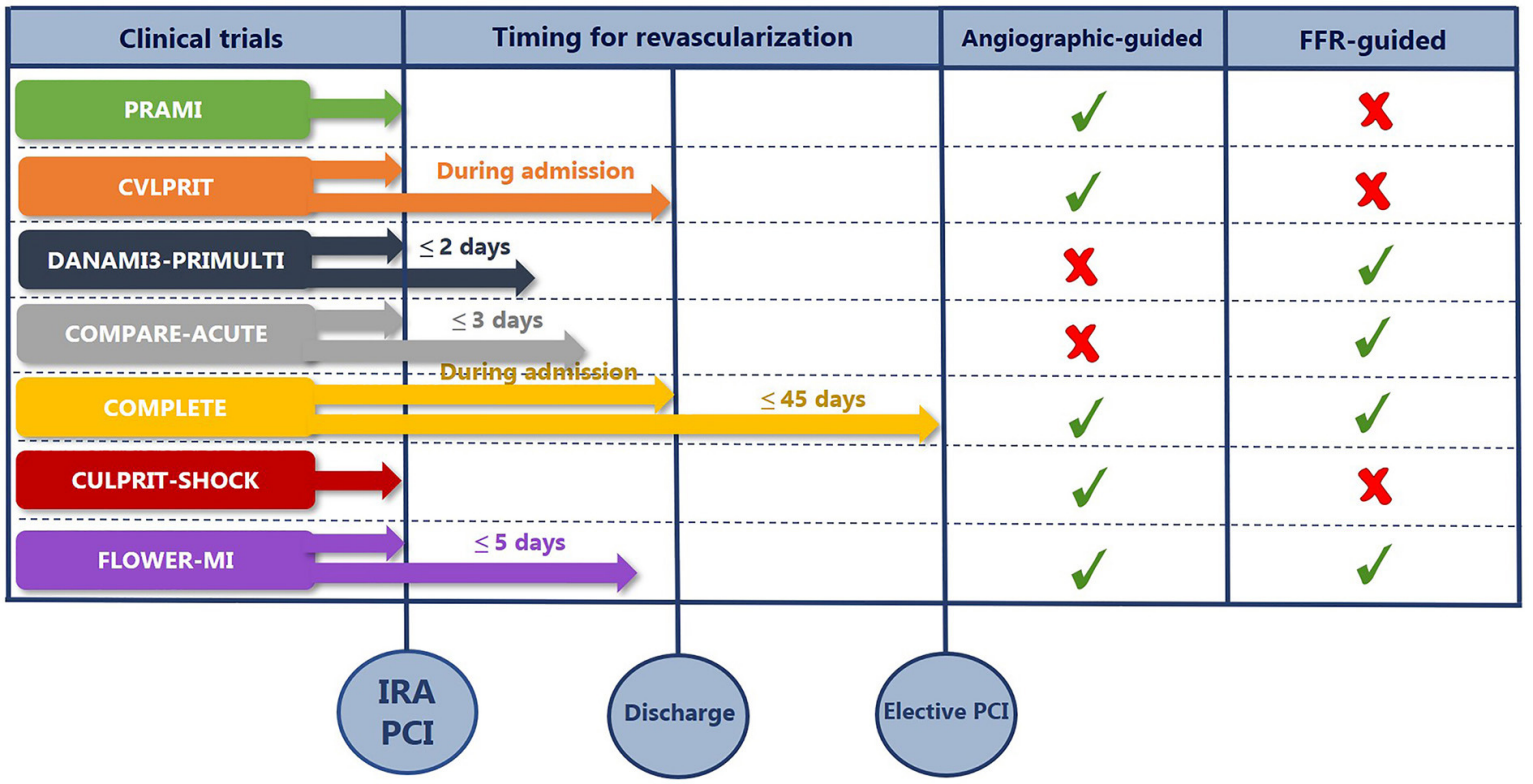
The summary of timing for complete revascularization and the method of guidance for PCI. COMPARE-ACUTE, Comparison Between FFR Guided Revascularization vs. Conventional Strategy in Acute STEMI Patients With MVD; COMPLETE, Complete vs. Culprit-Only Revascularization Strategies to Treat Multivessel Disease after Early PCI for STEMI; CULPRIT-SHOCK, Culprit Lesion Only PCI vs. Multivessel PCI in Cardiogenic Shock; CVLPRIT, Complete vs. Lesion-only Primary PCI; DANAMI3-PRIMULTI, Primary PCI in Patients With ST-elevation Myocardial Infarction and Multivessel Disease: Treatment of Culprit Lesion Only or Complete Revascularization; FFR, fractional flow reserve; FLOWER-MI, FLOW Evaluation to Guide Revascularization in Multi-vessel ST-elevation Myocardial Infarction IRA, infarct-related artery; PCI, percutaneous coronary intervention; PRAMI, Preventive Angioplasty in Acute Myocardial Infarction.

Figure 1 summarizes the timing of complete revascularization from the major RCTs in STEMI patients with MVD and the method of guidance for PCI.

#### Medication for plaques stabilization

Despite the impact of revascularization on the non-culprit lesion, optimal medical therapy is still mandatory. In brief, statins can stabilize the coronary artery vulnerable plaques and able to reduce the plaque volume (42–44). The proprotein convertase subtilisin kexin type 9 (PCSK9) inhibitor also alters the plaque morphology in ACS patients (45). Consequently, these medications prevent future adverse events for ACS patients.

## The current recommendations

The 2021 ACC/AHA Guideline for Coronary Artery Revascularization does not recommend routine CR for CS patients with complicated AMI, including STEMI and Non-ST segment elevation acute coronary syndrome (class of recommendation (COR) 3: Harm) (32). This recommendation is in contrast to the 2017 European Society of Cardiology (ESC) STEMI guidelines that suggest considering non-IRA PCI in patients with cardiogenic shock (COR: 2a) (1). For patients whose CS persisted after PPCI of the culprit lesion, the 2016 U.S. Appropriate Use Criteria for Coronary Revascularization in Patients With Acute Coronary Syndromes stated that it is appropriate to perform an immediate non-IRA PCI (46). For low-risk patients with residual, non-complex lesions, immediate PCI of the non-IRA lesions during the index procedure might be considered (COR: 2b).

Another core recommendation from the ACC/AHA guideline is to defer non-IRA PCI after successful PPCI of the IRA. For non-IRA lesions that are suitable for revascularization, staged PCI to achieve CR is recommended to reduce combined CV death and MI. The staged PCI should be performed either during the index hospitalization or within 45 days after the PPCI (COR: 1). In contrast, the ESC guideline suggests performing staged PCI before hospital discharge (COR: 2a). However, it should be noted that the 2017 ESC guidelines were published prior to the results of the CULPRIT-SHOCK trial and the COMPLETE trial. For residual complex lesions, the heart team approach is recommended (COR: 1) and coronary artery bypass graft (CABG) is a reasonable approach for revascularization to reduce CV events (COR: 2a).

**Figure 2:**
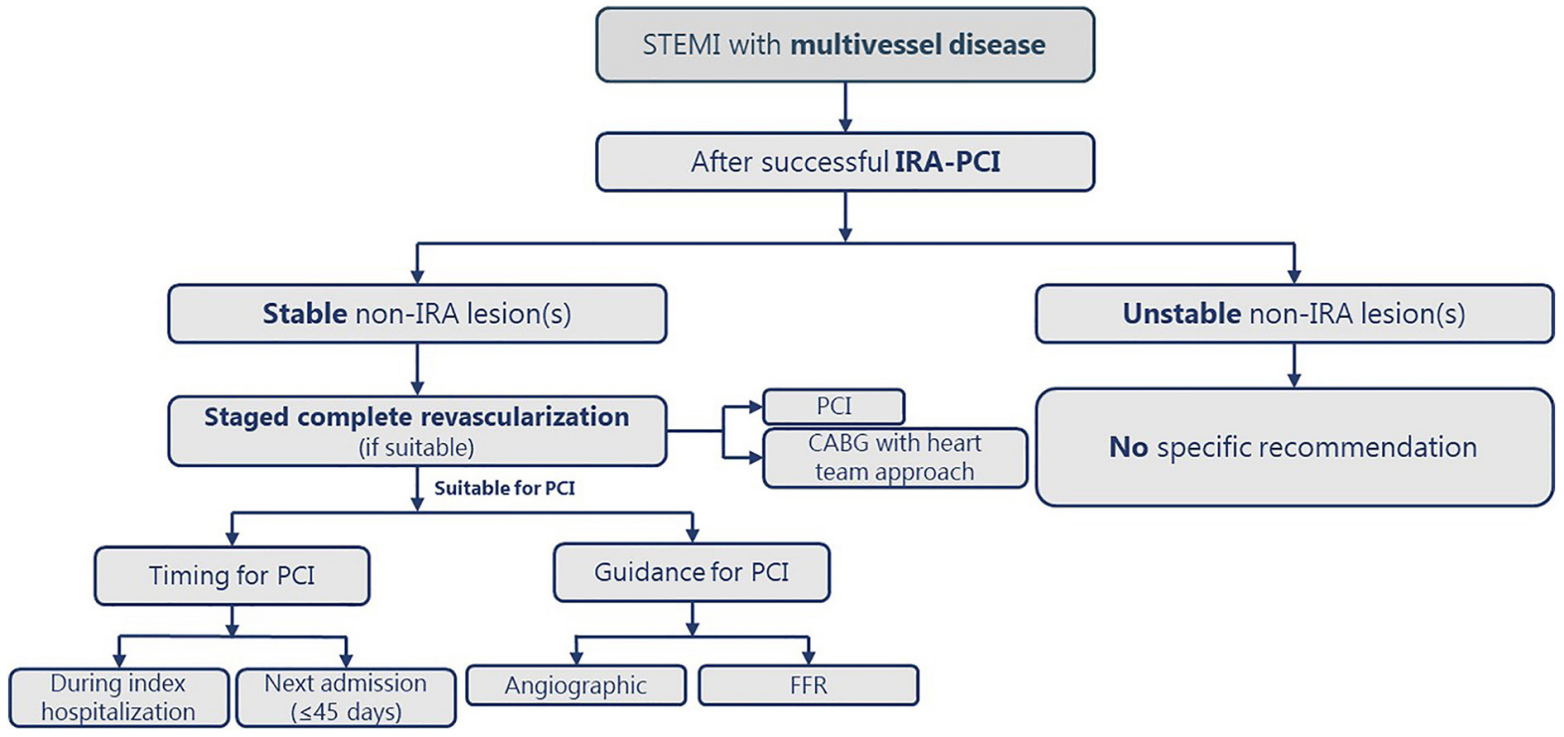
The practical approach for complete revascularization in patients with STEMI and MVD. CABG, coronary artery bypass graft; CS, cardiogenic shock; FFR; fractional flow reserve; IRA, infarct-related artery; MVD, multivessel disease; PCI, percutaneous coronary intervention; STEMI, ST-segment elevation myocardial infarction.

Figure 2 illustrates a practical approach for revascularization in STEMI with MVD patients.

## Areas of uncertainty and future directions

There are some areas of uncertainty. First, the revascularization strategies for CS complicate STEMI with residual unstable non-IRA lesions. In the CULPRIT-SHOCK trial, the residual lesion is confined to stable non-IRA lesions. Second, there is currently no consensus regarding the optimal timing of the staged procedure. Finally, the best method for guiding revascularization for non-culprit lesions: angiographic-guided, intravascular imaging-guided, or FFR-guided for CR, remains unclear. Moderate-to large-scale RCTs are needed to answer these questions.

## Conclusions

The best approach for STEMI with MVD is to aim for complete revascularization. Initially, perform PPCI of the IRA to gain the survival benefits, followed by routinely-staged revascularization of stable, non-IRA lesions to minimize future adverse clinical events. The staged PCI should be performed either during the index admission or not more than 45 days after the index procedure, according to local practices and resources. Either angiographic or FFR guidance for revascularization could be used. For patients with complex lesions that are not suitable for PCI, CABG applying the heart team approach would be indicated. Finally, the benefits of revascularization should be weighed against the additional risks from the procedure.

## Author contributions

KT contributes to all aspects of this manuscript.

## Conflict of interest

The author declares that the research was conducted in the absence of any commercial or financial relationships that could be construed as a potential conflict of interest.

## Publisher's note

All claims expressed in this article are solely those of the authors and do not necessarily represent those of their affiliated organizations, or those of the publisher, the editors and the reviewers. Any product that may be evaluated in this article, or claim that may be made by its manufacturer, is not guaranteed or endorsed by the publisher.

## Glossary

ACC, American College of Cardiology; AHA, American Heart Association; CABG, coronary artery bypass graft; CI, confidence interval; COMPARE-ACUTE, The Comparison Between FFR Guided Revascularization vs. Conventional Strategy in Acute STEMI Patients With MVD trial; COMPLETE; The Complete vs. Culprit-Only Revascularization Strategies to Treat Multivessel Disease after Early PCI for STEMI trial; COR, class of recommendation; CR, complete revascularization; CS, cardiogenic shock; CTO, chronic total occlusion; CULPRIT-SHOCK, Culprit Lesion Only PCI vs. Multivessel PCI in Cardiogenic Shock trial; CVLPRIT, The Complete vs. Lesion-only Primary PCI trial; EXPLORE, Evaluating Xience and Left Ventricular Function in Percutaneous Coronary Intervention on Occlusions After ST-Segment Elevation Myocardial Infarction; DANAMI3-PRIMULTI, The Primary PCI in Patients With ST-elevation Myocardial Infarction and Multivessel Disease: Treatment of Culprit Lesion Only or Complete Revascularization trial; DAPT, dual antiplatelet therapy; DEFINE-FLAIR, Functional Lesion Assessment of Intermediate Stenosis to Guide Revascularisation; ESC, European Society of Cardiology; FFR, fractional flow reserve; FLOWER-MI, FLOW Evaluation to Guide Revascularization in Multi-vessel ST-elevation Myocardial Infarction trial; HR, hazard ratio; IDN, ischemia-driven revascularization; iFR-SWEDEHEART, Instantaneous Wave-Free Ratio vs. Fractional Flow Reserve in Patients With Stable Angina Pectoris or Acute Coronary Syndrome; IRA, infarct-related artery; LMCA, left main coronary artery; LVEDD, left ventricular end-diastolic volume; LVEF, left ventricular ejection fraction; MACCE, major adverse cardiac and cerebrovascular events; MACE, major adverse cardiac events; MI, myocardial infarction; MVD, multivessel disease; PCI, percutaneous coronary intervention; PPCI, primary percutaneous coronary intervention; PRAISE, prediction of adverse events following an acute coronary syndrome; PRAMI, The Preventive Angioplasty in Acute Myocardial Infarction trial; PROSPECT, The Imaging Study in Patients With Unstable Atherosclerotic Lesions; RCTs, randomized controlled trials; REDUCE-MVI, Reducing Micro Vascular Dysfunction in Revascularized STEMI Patients by Off-target Properties of Ticagrelor trial; RR, relative risk reduction; STEMI, ST-segment elevation myocardial infarction; SYNTAX, Synergy between PCI with Taxus and Cardiac Surgery.
